# Natural Polysaccharides as Preventive and Therapeutic Horizon for Neurodegenerative Diseases

**DOI:** 10.3390/pharmaceutics14010001

**Published:** 2021-12-21

**Authors:** Manel Dhahri, Mawadda Alghrably, Hamdoon A. Mohammed, Syed Lal Badshah, Noreen Noreen, Fouzi Mouffouk, Saleh Rayyan, Kamal A. Qureshi, Danish Mahmood, Joanna Izabela Lachowicz, Mariusz Jaremko, Abdul-Hamid Emwas

**Affiliations:** 1 Biology Department, Faculty of Science Yanbu, Taibah University, Yanbu El-Bahr 46423, Saudi Arabia; manel.dhahri@gmail.com; 2 Division of Biological and Environmental Sciences and Engineering (BESE), King Abdullah University of Science and Technology (KAUST), Thuwal 23955, Saudi Arabia; mawadda.alghrably@kaust.edu.sa (M.A.); Mariusz.jaremko@kaust.edu.sa (M.J.); 3 Department of Medicinal Chemistry and Pharmacognosy, College of Pharmacy, Qassim University, Buraydah 51452, Saudi Arabia; ham.mohammed@qu.edu.sa; 4 Department of Pharmacognosy, Faculty of Pharmacy, Al-Azhar University, Cairo 11371, Egypt; 5 Department of Chemistry, Islamia College University, Peshawar 25120, Pakistan; shahbiochemist@gmail.com (S.L.B.); noreenkhattak777@gmail.com (N.N.); 6 Department of Chemistry, Faculty of Science, Kuwait University, Safat 13060, Kuwait; fmouffouk@gmail.com; 7 Chemistry Department, Birzeit University, Birzeit P627, Palestine; sarayyan@birzeit.edu; 8 Department of Pharmaceutics, Unaizah College of Pharmacy, Qassim University, Unaizah 51911, Saudi Arabia; ka.qurishe@qu.edu.sa; 9 Department of Pharmacology and Toxicology, Unaizah College of Pharmacy, Qassim University, Unaizah 51911, Saudi Arabia; ma.alam@qu.edu.sa; 10 Department of Medical Sciences and Public Health, Università di Cagliari, Cittadella Universitaria, 09042 Monserrato, Italy; 11 Core Labs, King Abdullah University of Science and Technology, Thuwal 23955, Saudi Arabia

**Keywords:** polysaccharides, neuroprotection, regulated cell death, neurodegenerative diseases, Parkinson’s disease, Alzheimer’s disease

## Abstract

Neurodegenerative diseases are a serious and widespread global public health burden amongst aging populations. The total estimated worldwide global cost of dementia was US$818 billion in 2015 and has been projected to rise to 2 trillion US$ by 2030. While advances have been made to understand different neurodegenerative disease mechanisms, effective therapeutic strategies do not generally exist. Several drugs have been proposed in the last two decades for the treatment of different types of neurodegenerative diseases, with little therapeutic benefit, and often with severe adverse and side effects. Thus, the search for novel drugs with higher efficacy and fewer drawbacks is an ongoing challenge in the treatment of neurodegenerative disease. Several natural compounds including polysaccharides have demonstrated neuroprotective and even therapeutic effects. Natural polysaccharides are widely distributed in plants, animals, algae, bacterial and fungal species, and have received considerable attention for their wide-ranging bioactivity, including their antioxidant, anti-neuroinflammatory, anticholinesterase and anti-amyloidogenic effects. In this review, we summarize different mechanisms involved in neurodegenerative diseases and the neuroprotective effects of natural polysaccharides, highlighting their potential role in the prevention and therapy of neurodegenerative disease.

## 1. Introduction

The prevalence of neurodegenerative pathologies increases significantly with growing life expectancy. Neurodegenerative diseases including common diseases such as Alzheimer’s disease (AD) and Parkinson’s disease (PD) impose a global public health burden. For instance, the number of AD patients in the USA alone is expected to rise from 5.8 million to 13.8 million by the mid-twenty-first century [1]. AD is the fifth leading cause of death among Americans age 65 and older, and the sixth leading cause of death in the USA in general [1]. In the USA alone, the total number of unpaid care hours given to people suffering from AD and other neurodegenerative diseases is estimated to be 18.6 billion hours—valued at nearly $44 billion [1].

Neurodegenerative diseases have heterogeneous symptoms, since they are linked to the portion of the brain affected, which can vary. These pathologies are typically associated with the accumulation of abnormal aggregated proteins that lead to progressive dysfunction of the central nervous system through various biological processes, such as oxidative stress and neuroinflammation [2]. Considering the complexity of the human brain, there is still a long way to go before all neurodegenerative disease mechanisms are deciphered. Moreover, the success of therapeutic strategies for neurodegenerative diseases including PD and AD is still limited. In fact, existing clinical drugs are not effective and only keep the patient’s health from degrading [3]. Furthermore, current treatments are linked to significant social, economic, and personal costs [4]. Intense research is focused on finding efficient drugs for neurodegenerative diseases. For example, the number of studies published containing the key words “Neurodegenerative disease” and “treatments” was 10,566 for the period 2015–2020, according to Scopus database (www.scopus.com, accessed on 27 November 2021).

In this context, natural products could play a leading role in the search for new drugs for the treatment of neurodegeneration [5,6,7]. Of note, more than 80 percent of drugs are of natural origin [8]. Natural polysaccharides (general formula of C_x_(H_2_O)_y_; where x is number 200_2500) occurs naturally in living matter (on the contrary to polysaccharides combined artificially in the process of organic synthesis) and principally play structural and storage functions. Natural polysaccharides can be classified according to their origin, namely plants (e.g., starch, cellulose), algae (e.g., agar, alginates), animals (e.g., chitin, hyaluronic acid), bacteria (e.g., dextran, polylactosamine), and fungal (e.g., chitosan, elsinan) [Torres, Fernando G., et al. “Natural polysaccharide nanomaterials: an overview of their immunological properties.” International journal of molecular sciences 20.20 (2019): 5092.].

As one of the most widely distributed biomolecules in nature, natural polysaccharides have received considerable attention in the last decade because of their diverse pharmacological activity as inhibitors of cellular processes, with their antioxidant, anticoagulant, antithrombotic and anticancer effects [9]. Polysaccharides from natural sources (plants, algae, animals, bacteria, fungal) have recently been used in many biomedical applications owing to their health-promoting and therapeutic efficacy [10]. Polysaccharides from marine organisms such as algae have been reported to have anti-inflammatory and neuroprotective effects, related to their antioxidant activity [11]. Furthermore, polysaccharides such as agar, alginates, and carrageenan, which are all obtained from macroalgae (seaweed), are used in drug formulation and drug delivery [12,13]. Polysaccharides are also used in the pharmaceutical and cosmetic industries and have nutraceutical applications due to their antioxidant activity. Recent studies have shown that natural polysaccharides have anti-neuroinflammatory [14], anticholinesterase [15,16], anti-amyloidogenic [14,16,17] and anti-neurotoxological [17,18] effects. In addition, numerous studies in vitro and in vivo have revealed that natural polysaccharides could prevent or inhibit neurological disorders. Some polysaccharides have been shown to stimulate neuroprotective effects in vivo, in different models for neurodegeneration [19]. Interestingly, a green algae polysaccharide was shown to have anti-PD activity through inhibition of α-Synuclein fibrillation [20,21]. Anti-PD activity was also recently determined for natural polysaccharides via increasing cellular autophagy [22]. Several other polysaccharides also have anti-AD effects [23,24,25,26,27]. Table 1 summarizes the potential protective and therapeutic activities of natural polysaccharides in neurodegeneration.

Natural macromolecules have interesting physicochemical characteristics such as a richness of functional groups that allow chemical modification and functionalization. Moreover, natural polysaccharides have other beneficial properties including solubility in water, biocompatibility, high stability and low toxicity [78]. This review highlights the neuroprotective effects of natural polysaccharides, providing a methodical basis for the development of new anti-neurodegenerative therapies based on natural polysaccharides. Here, we describe the inhibitory role of polysaccharides in multiple molecular process involved in neurodegeneration. We present also numerous examples of natural polysaccharides, which have been proved to slow down the neurodegeneration or were even examined as a potential cure for neurodegenerative diseases.

## 2. Polysaccharide’s Treatment in Neurodegenerative Disorders

Neurodegeneration is a complex process leading to neuron dysfunction. Numerous intracellular and extracellular mechanisms, involving different biochemical pathways, lead to neurodegeneration in additive and/or synergistic manner, and all together must be considered when we are looking for an effective prevention and therapy of neurodegenerative diseases.

In this section, we describe briefly different processes, which significantly affects neurodegeneration, and inhibitory activity of natural polysaccharides that could be used in the prevention and/or therapy of neurodegenerative disorders. According to the recent studies, the processes of amyloid formation, uncontrolled reactive oxygen species (ROS) production, mitochondrial dysfunction, misfunctioning of cholinesterase, and ferroptosis influences the most neurodegeneration. All this events, together and/or separately lead to uncontrolled oxidation and inflammation within neurons, which finishes with deterioration of neurons in nervous system. Natural polysaccharides which prevent and/or interfere with processes that initiate neurodegeneration could be used in preventive therapy. Those molecules that can reverse neurodegeneration and ameliorate cognitive, learning, memory and motorial impairment could be considered for healing therapy.

### 2.1. Mechanisms of Neurodegenerative Disease

Oxidation is a physiological process, and ROS are crucial in several processes, for instance cell signaling. However, excessive and uncontrolled ROS production lead to serious complications and development of neurodegenerative diseases such as PD and AD [79]. In addition, decrease of the antioxidant molecules, such as glutathione and antioxidant enzymes, are linked to the age-associated impairments. The presence of high levels of oxidised proteins, advanced glycation end-products, lipid peroxidation end-products, oxydising species (e.g., peroxides), and oxidative modifications in nuclear and mitochondrial DNA have been found in nervous system of AD patients [80]. Overall oxidative stress conditions lead to the brain lipids oxidation and has an established role in the development and progression of the neurodegenerative disorders [81,82].

Mitochondrial dysfunctions has been widly documented in AD-relation [83,84].The intracellular production of ROS mainly takes place in the mitochondria during electron transfer processes [85]. In addition, oxidative phosphorylation is responsible for the endogenous production of about 90% of ROS [86]. Unfortunaly, broken mitochondria produce and release excessive ROS, and decrease oxidative metabolism enzymes (e.g., α-ketoglutarate dehydrogenase complex, pyruvate dehydrogenase complex, and cytochrome oxidase). Moreover, calcium dyshomeostasis was found in the mitochondrial dysfunction, and was linked to AD development and progression [86].

Several salient and established features of neurodegenerative diseases (e.g., lipid peroxidation and iron dyshomeostasis) are consistent with ferroptosis [87]. Ferroptosis is defined by Nomenclature Committee on Cell Death as “regulated cell death (RCD) initiated by oxidative perturbations of the intracellular microenvironment that is under constitutive control by Glutathione Peroxidase 4 (GPX4) and can be inhibited by iron chelators and lipophilic antioxidants” [88]. The molecular processes and morphological changes in the cell during ferroptosis are distinct from that of apoptosis and other known RCD. Of note, ferroptosis depends on iron ions, which in excess of its ionic free form promote non-enzymatic lipid oxidation via lysosomal Fenton reactions [89]. Conversely to other known RCD process, ferroptosis can be turned back if the iron chelators and lipophilic antioxidants are delivered on time. Morphologically, ferroptosis manifests with a necrotic morphotype (with a predominance of mitochondrial alterations encompassing shrinkage, an electron-dense ultrastructure, reduced/disappeared cristae, and ruptured outer mitochondrial membrane). Of note, neurodegenerative diseases are often accompanied by degeneration of mitochondrial activity. Mitochondrial damage has been found to be involved in lipid peroxidation and iron dyshomeostasis in neurodegenerative diseases [87].

The role of neuroinflammation in neurodegeneration has been widly described [90,91]. In particular, AD intensity and progression have been linked to the immunological brain mechanisms including misfolded proteins and release of the inflammatory mediators [92]. Moreover, systemic inflammatory mediators and modulators, e.g., cytokines, chemokines, caspases, Prostanoids, neuroprotectin D1, Nitric oxide (NO), and ROS are probably interfering with the brain immunological processes and further promote AD disease progression [92]. Several other causes linked to the neuroinflammation might contribute to the development and progression of AD, and other neurodegenerative diseases.

Acetylcholinesterases (AChE) are essential for the proper functioning of the nervous system. They belong to the cholinesterases (ChEs) family, which has a specific carboxylic ester hydrolase function that metabolizes choline esters. There are two types of cholinesterases: AChE and butyrylcholinesterase (BChE). BChE is a serum cholinesterase or pseudo-cholinesterase, and it is a less-specific enzyme that utilizes butyrylcholine as a substrate. AChE hydrolyzes and inactivates the key chemical signaling molecule—acetylcholine (ACh), which controls the transmitter’s concentration at the synapse. AChE is present in the central nervous system (CNS) at cholinergic synapses and neuromuscular junctions. It breaks synaptic transmission by inactivating Ach, which is released by cholinergic nerve endings and critical for normal functioning of the central and peripheral nervous systems [93]. Changes in ACh levels or the number of cholinergic receptors and activity in specific areas of the nervous system have been linked to various neurodegenerative diseases, including AD and PD, Huntington’s disease (HD). The cognition-related, mental, and motor disorders associated with these diseases have been linked to cholinergic circuit dysfunction. Neurodegenerative disorders, including AD and others, are often affected by cholinergic system degeneration. AD pathogenesis is not well understood, whereas the only valid hypothesis that has been accepted is a deficiency in acetylcholine, a neuromodulator. High acetylcholinesterase activity is linked with cholinergic dysfunction and cognitive deterioration in AD, due to acetylcholine’s rapid hydrolysis. As a result, inhibiting acetylcholinesterase enhances acetylcholine availability, which boosts synaptic transmission and memory function. Additionally, ACh’s role as a modulator of inflammation—both inside and outside the nervous system—has been associated with multiple sclerosis [94].

Protein folding is the process by which protein assumes its native three-dimensional structure and becomes biologically functional. Protein folding is thus pivotal for human health and wellbeing [95]. Protein misfolding results in the formation of oligomers and deposits that cause numerous diseases, including AD, PD, HD, transmissible spongiform encephalopathies (TSEs), amyotrophic lateral sclerosis (ALS) and type II diabetes mellitus. Disease develops due to the presence of disease-specific misfolded and aggregated proteins or peptides [96,97,98,99,100,101,102,103]. There are more than 50 human diseases that are now associated with protein aggregation [104,105,106]. Numerous studies have revealed a common pathogenic mechanism associated with these neurodegenerative disorders; the aggregation of misfolded protein in various regions of the brain, contributing to central nervous system amyloidosis [99,107]. To describe the processes of amyloid formation, multiple models have been used to date. Of particular interest are the major mechanistic pathways that have been extensively described by Ghosh and De [108]. These authors explain aggregation through five mechanisms including: (1) aggregation via self-association of monomeric protein, (2) aggregation through conformationally altered protein, (3) nucleation-dependent mechanisms, (4) nucleation-independent mechanisms and (5) aggregation through direct chemical linkages, chemical degradation, and other pathways.

The first model (1) is called aggregation via self-association of monomeric protein, in which the addition of monomers leads to the formation of aggregated proteins [109]. In other words, monomers of the protein of interest have a high tendency to self-assemble via different interactions such as electrostatic or hydrophobic forces. This leads to the formation of oligomers, a reversible event [108]. Over time, these reversible oligomers convert into irreversible protein aggregates through the formation of covalent bonds such as disulfide bonds.

The second model (2) is aggregation through conformationally altered protein. In this model, aggregation occurs by proteins that have a conformationally altered or partially unfolded state, which appears to result in them forming oligomers more frequently [109]. In the initial stage, this process requires external stimuli such as heat or shear [109]. There are a few proteins, however, that follow this mechanism in order to undergo aggregation.

Most protein aggregation happens through the nucleation-dependent mechanism, which is the third model (3) of aggregation. This mechanism follows a sigmoidal curve and goes through three consecutive phases [110]:

(a) The lag/nucleation phase—the time needed to form nuclei. Depending on the solution conditions, the duration of this phase will range from minutes to several days [110].
(b) The elongation phase—fibril development begins directly after the formation of nuclei, by further association of monomers or oligomeric species with the nuclei, and ends with the formation of ordered fibrils known as protofibrils [108]. As this stage contributes to the development of more stable protofibrils, this process is regarded as a thermodynamically favorable step [110].
(c) The saturation phase—monomer concentration becomes remarkably low or almost stable during the saturation process, and protofibrils combine with each other to develop mature amyloid fibrils. In the steady-state phase, a dynamic equilibrium exists between fibrillar structures and monomeric proteins [108].

The fourth aggregation model (4) is a nucleation-independent process that involves the creation of linear multimers/spherical oligomers. This model is described by a multi-step reaction with equal rate constants (k), following an exponential polymerization curve [108]. The process proceeds downhill towards polymerization as soon as the accumulation happens [111]. A variety of energetically favorable phases are included in this aggregation pathway, whereby amyloidogenic monomers are added to the growing aggregated particles [111].

The last aggregation process (5) is through direct chemical linkages, chemical degradation, and other pathways. Many proteins are aggregated through chemical bonds, such as intermolecular disulfide bond formation [112]. Cysteine residues that are found on the protein surface have a greater ability to engage in disulfide bond formation than those present in the inner core [112]. In addition, aggregation takes place through disulfide exchange via β-elimination in the absence of free cysteine. Non-disulfide cross-linking, on the other hand, may also promote protein aggregation [113,114,115]. Nevertheless, disulfide bond formation/exchange reactions, happen more often than non-disulfide cross-linking reactions. In addition, many mechanisms of chemical degradation, such as glycation and dimerization, may induce protein peptides to aggregate [116,117]. In addition to modifying protein secondary or tertiary structure, chemical degradation influences protein hydrophobicity, promoting protein unfolding [108]. Increasing data indicates that hydrophobic-hydrophilic interfaces/surfaces also have a primary role in promoting the tendency of amyloidogenic peptides and proteins to accumulate in vitro. Campioni et al. [118] found that the existence of an air-water interface can dramatically influence the development and elongation of fibrils of α-Synuclein. It is also very important to consider interfacial effects as they have various roles in the aggregation of α-synuclein, regulating primary and secondary nucleation and elongation processes. In addition, Li et al. [119] showed that after unlocking S-S bonds with a reducing agent—tris(2-carboxyethyl) phosphine (TCEP)—globular proteins with strong aggregation propensity and an α-helical rich structure can easily undergo superfast amyloid-like aggregation. The oligomeric species as well as protofibrils are produced within a few minutes after activation of rapid amyloid-like aggregation, which further contributes to the formation of a macroscopic nanofilm at the air/water interface and microparticles in bulk solution. This unique approach offers a rational design approach for the practical production of amyloid products. Therefore, prior to developing an efficient method for inhibition of protein aggregation, it is important to investigate the precise causes of protein aggregation [108].

Neurotoxins are substances that cause extensive damage in central and peripheral nervous system. Neurotoxins are a large family of exogenous chemicals that can negatively impact functioning of the mature and premature nervous system. Often neurotoxins are destructive to nervous tissue, due to their capacity to explicitly target neural components. Common examples of these neurotoxins include: ethanol, manganese glutamate, lead, nitric oxide (NO), tetanus toxin, botulinum toxin (e.g., Botox) and tetrodotoxin [120]. Certain chemical structures such as glutamate and nitric oxide are essential for the proper function of the body, but at high concentrations become neurotoxic. A series of investigations has shown that PD onset correlates with certain environmental and genetic risk factors, such as toxin exposure and mutations in specific genes [121]. For instance, 6-hydroxydopamine (a toxic metabolite of dopamine) has been identified in the urine and brains of patients with PD [122]. This neurotoxin can selectively enter dopaminergic neurons via noradrenaline or dopamine transporters (NAT and DAT, respectively), followed by the overproduction of ROS through enzymatic oxidation [123]. Therefore, the increased concentration of ROS elicited by 6-hydroxydopamine, damages fundamental cellular structure, and causes dopaminergic neuron cell death and motor complications in PD patients [124].

### 2.2. Neuroprotective and Therapeutic Effects of Natural Polysaccharides

#### 2.2.1. Anti-Amyloidogenic Effects of Polysaccharides

One of the major neuropathological hallmarks of AD is irregular folding and aggregation of amyloid-β protein (Aβ). The discovery of new Aβ aggregation inhibitors, which could be used in prevention and treatment, is however a recent development [125]. Ulvan, an acidic green macroalgal polysaccharide of the genus Ulva, has been documented by Liu et al. [125] to inhibit Aβ fibrillation as measured by fluorescence microscopy. Ulvan was revealed to inhibit Aβ fibrillogenesis in a concentration-dependent manner and to dynamically inhibit the development of A11-reactive Aβ oligomers, the most toxic species of Aβ [125]. Circular dichroism showed that ulvan blocks Aβ40′s conformation transition from the initial random coil to a β-sheet structure, but only delays Aβ42′s conformation transition [125]. Ulvan has also been found to substantially reduce the cytotoxicity of Aβ, measured by the 3-(4,5-dimethylthiazol-2-yl)-2,5-diphenyltetrazolium bromide (MTT) assay [125]. It also effectively reduces intracellular ROS levels and protects PC12 cells from the damage caused by Aβ fibrillation [125]. In addition, ulvan disaggregates preformed mature fibrils into off-pathway oligomers and substantially decreases their associated cytotoxicity [125]. The above findings not only thoroughly explain the inhibitory effect of ulvan on Aβ fibrillation and its associated cytotoxicity, but also provide new ideas for the production of usable seaweed food ingredients for the treatment of AD [125].

Different amyloid fibrils are strongly associated with several neurodegenerative conditions and are produced by the accumulation of internally disordered and inappropriately folded proteins [126,127,128]. Therefore, the development of compounds that could bind and prevent amyloid development is important [128]. In this respect, the activity of two sulfated polysaccharides against Aβ40 peptide aggregation has been investigated. Both chitosan (CHT) and its derivative N-trimethyl chitosan chloride (TMC) had specific inhibitory action against the fibrillogenesis of Aβ40 as measured microscopically [128]. Their inhibitory mode consist in formation of electrostatic linkages between the positively charged CHT/TMC compounds and the negatively charged Aβ40 units [128]. Stronger preventative behavior of TMC compared to CHT indicated the importance of the polymeric chain’s charge density in the prevention of fibril development [128]. Molecular docking and simulation also showed potential linkages of CHT/TMC with Aβ40 on the atomic level, showing that Aβ40 is a stabilized unit after electrostatic linkages with both charged CHT and TMC amines respectively. The linkage of these polysaccharides with the essential Aβ40 peptide hydrophobic central region might account their ability to prevent nuclear spread of fibrillary structures [128]. Current findings indicate that integration into the polymer structural template of sugars like D-glucosamine and N-trimethyl-D-glucosamine may be a novel method for the production of new anti-amyloid molecules.

Some naturally occurring polysaccharides show anti-amyloidogenic effects through their inhibition of protein fibrillation and dissolution of protein fibrils. For example, *Chlamydomonas reinhardtii* sulfated polysaccharides (with a sulfate content of around 29.4%) were examined for their potential inhibition of α-Synuclein fibrillation associated with PD, and could be potentially used in preventive therapy. The isolated sulfated polysaccharides efficiently inhibited α-Synuclein fibrillation. In addition, soluble protein was observed by odium dodecyl sulphate-polyacrylamide gel electrophoresis gel-imaging after complete fibrillation of α-Synuclein [20]. Panigrahi, Gitanjali P., et al. isolated semi-purified sulfated polysaccharides named Cr-SPS from *Chlamydomonas reinhardtii* containing 34% sulfate. The Cr-SPS inhibited fibrillation of α-Synuclein familial mutants A30P, A53T and E35K, and artificial mutants E46K and E57K, and increased solubilized α-Synuclein. The effects of Cr-SPS make them potential therapeutic agents for protein aggregation disorders including PD [21]. Similarly, the disappearance of β-amyloid peptide fibrils has been demonstrated for sulfated polysaccharides isolated from *Ecklonia maxima*, *Gelidium pristoides* and *Ulva rigida* [15]. These sulfated polysaccharides also inhibited the aggregation of amyloid fibrils compared to control untreated peptide [15]. Furthermore, heparan sulfate proteoglycan linear polysaccharides bind to the amyloid structure and to the amyloid precursor protein/peptide [129]. Kisilevsky and Walter synthesized novel glycosaminoglycan anti-amyloid compounds as precursors for heparan sulfate, to alter its structure and inhibit its amyloid precursor protein/peptide-binding and fibril inducing properties [129,130].

Zhou et al., suggested that polysaccharides (LBP1) derived from *Lycium barbarum* can reduce Aβ levels and increase cognitive functions in a APP/PS1 transgenic mouse [33]. Thus, could be used in prevention and therapy of AD. LBP1 can enhance neurogenesis as measured by BrdU/NeuN double labelling [33]. It can also restore synaptic dysfunction in the hippocampal CA3-CA1 pathway. Furthermore, in vitro cell assays show that Aβ processing may be affected by LBP1 [33]. PD is caused by aggregation of the presynaptic protein α-Synuclein. Various medications exist to treat PD but are not very promising in their inhibition of disease progression and have several side effects. Choudhary et al. [20], investigated the effect of sulfated polysaccharides extracted from *Chlamydomonas reinhardtii* on α-Synuclein fibrillation using both microscopic and spectroscopic approaches [20]. By measurement of α-Synuclein fibrillation kinetics, it was demonstrated that these polysaccharides are successful in preventing fibrillation. Electrophoresis revealed the presence of soluble protein in the presence of polysaccharides [20]. Fibrillation-related morphological changes were tracked by microscopy and suggested the polysaccharides attach effectively to α-Synuclein and thus postpone transformation of α-helical structures into β-pleated sheets [20]. These polysaccharides are still effective after the onset of α-Synuclein fibrillation through their ability to relax pre-generated fibrils [20]. These finding suggest that algal polysaccharides could act as alternate preventive treatments for PD and several disorders associated with protein aggregation [20]. PD-associated glutamate and alanine residue mutations of the α-Synuclein protein cause unique tertiary interactions that are important to maintain this protein in a stable native condition and cause more aggregation. Several commonly used medications for the treatment of PD are ineffective and have side effects associated with them [20]. Previous studies on marine algae containing sulfated polysaccharides revealed various medicinal properties. Panigrahi et al. [21] isolated Chlamydomonas reinhardtii sulphated polysaccharides (Cr-SPs) and studied their effects on the suppression of fibrillation/aggregation of α-Synuclein mutants with several microscopic and spectroscopic methods [21]. Measurement of α-Synuclein fibrillation kinetics showed that these polysaccharides can sufficiently suppress α-Synuclein mutant fibrillation [21], and could be potentially used in preventive therapy of PD. Microscopy was used extensively to examine morphological variations associated with fibrillation/aggregation of α-Synuclein. After completion of the fibrillation/aggregation process, electrophoretic results showed these polysaccharides enhance the total quantity of soluble protein [21]. Circular dichroism related techniques revealed that Cr-SPs are slow converters of native protein into accumulated β-sheet structures. Therefore, the referenced work offers insight into why Cr-SPs may prevent PD and other disorders related to protein aggregation, with important medicinal benefits.

#### 2.2.2. Antioxidant Activity of Polysaccharides

The exact mechanisms by which polysaccharides have antioxidant activity are unestablished yet, however some hypotheses have been suggested in the literature [131,132,133,134,135]. Various factors could be important in natural polysaccharide antioxidant potency; including structural properties, chemical composition, type of the molecular linkages and molecular weight, as well as the extraction process used to obtain the polysaccharides form its natural source [131,132,133].

The antioxidant activity of natural polysaccharides is influenced by factors including; the number of hydroxyl groups and presence of carboxylic acids, sulfate content, sulfate attachment position, and molecular weight [10,134,135]. For example, low molecular weight chitosan (9 kDa) scavenges superoxide radicals better than high molecular weight chitosan (760 kDa), with 85.8% and 35.5% inhibition respectively [136]. Higher sulfate content is regularly associated with higher antioxidant activity in polysaccharides [137,138], as shown by Shao et al., in natural marine derived polysaccharides [139]. Additionally, sulfated lower molecular weight polysaccharides of Ulva pertusa show stronger antioxidant activity than the sulfated higher molecular weighted plant polysaccharides [135]. Lower molecular weight and higher uronic acid content was associated with better antioxidant activity in *Chimonobambusa quadrangularis* freeze-dried polysaccharide residue [140].

One proposed polysaccharide antioxidant mechanism for glycone is that it is usually present in natural sources, which contain other non-sugar aglycone moieties, such as polyphenols, flavonoids, lipids, amino acids, and nucleic acids. However, the aglycone (non-sugar part) has the primary antioxidant activity of sugar-aglycone combinations. Evidence for this comes from tea leaf polysaccharides, whereby crude tea leaf polysaccharides show more antioxidant activity than semi-purified tea polysaccharides, due to the presence of epigallocatechin gallate polyphenol in the crude tea leaf mixture [141]. The ratio of polysaccharides to aglycone in polysaccharides aglycone mixtures could be a parameter influencing their activity [142,143,144]. For example, polysaccharide:protein ratio was found to affect free radical scavenging activity, and higher protein:polysaccharide ratio has better free radical scavenging activity for polysaccharide-protein complexes obtained from Ganoderma and Grifola. Studies have also confirmed that protein free samples of polysaccharide show no antioxidant activity [144]. The nature of the aglycone part of the polysaccharide non-sugar conjugations plays a significant role in the antioxidant potential of the whole mixture. Protein-free and protonated phenolic acid arabinoxylan polysaccharide mixtures have been evaluated for free radical scavenging activity. They showed higher antioxidant activity of the free protein phenolic acid arabinoxylan mixture over the protein containing mixture, indicating a role for phenolic acids in the antioxidant activity of sugar mixtures [145].

According to Bai et al. [26] the edible and curative mushroom, Maitake, is highly nutritious and contains a large amount of biologically active and health-promoting compounds [26]. A Maitake-derived polysaccharide called proteo-β-glucan (PGM), was reported to be a strong immunomodulator [26]. However, it remained uncertain if this polysaccharide could have immunomodulatory and neuroprotective effects on transgenic APP/PS1 mice, a common model for AD [26]. This study showed PGM-enhanced learning and memory, with reduced histopathological irregularities and neuronal loss in APP/PS1 mice [26]. Treatment with PGM might stimulate microglial cells and encourage the stability of microglial cells in Aβ-related plaques. Furthermore, PGM might strengthen Aβ phagocytosis, thus alleviating the strain of Aβ and the pathological changes in these experimental mice in the hippocampus and cortex [26]. In addition, PGM had no important impact on the body mass of the mice. Conclusively, this work suggests that PGM intake ameliorates memory decline through immunomodulation. Thus, dietary intake of PGM could be beneficial to ameliorate the effects of brain aging. In addition, Aβ can, through mitochondrial dysfunction, induce oxidative neuronal cell death [26]. Sirin et al. [146] investigated the possibility that exopolysaccharides (EPSs) originating from the *Lactobacillus delbrueckii subsp. bulgaricus* B3 and *Lactobacillus plantarum* GD2, protect SH-SY5Y cells from the apoptotic activity of Aβ1-42. EPSs depolarized mitochondrial membrane potential and decreased the apoptotic activity of Aβ1-42 in a concentration-dependent manner. These results led to the addition of EPSs to traditional medicine recommendations for different neurological disorders [146].

Park et al. [147] examined the impact of fucoidan and polyphenol extracts from *Ecklonia cava*, a brown marine algae, on cognitive function. Fucoidan and its polyphenol—in a specific ratio—enhanced learning and memory as compared to polyphenolic extract in various cognitive tests such as the Y-maze and the Morris water maze [147]. Tau hyper phosphorylation and amyloid-β were also down regulated [147]. In view of these outcomes, fucoidan-rich substances in macroalgae could be a potential material for improving cognitive function compared to polyphenol extract [147]. Alghazwi et al. [148] investigated the chemical composition of extracts from the brown macroalgae *Ecklonia radiata* in several in vitro neuroprotective assays. A total of six fractions were investigated to determine their action against oxidative stress and Aβ1-42 in neuronal cells [148]. These fractions were: crude extract (CE), polysaccharide (PS), phlorotannin (PT), high molecular weight (HM), low molecular weight (LM) and free sugar (FS). All fractions except HM prevented Aβ1-42 aggregation. They also displayed antioxidant properties against hydrogen peroxide-induced toxic effects. This study highlights the potential for enhancing neuroprotective effects with E. radiata brown seaweed components [148]. To enhance neurological function, these extracts may possibly be used as functional food or dietary supplements [148].

Habaike et al. [149] aimed to identify the protective roles of various components in Fomes officinalis Ames polysaccharides (FOAPs) in neuronal cells. Various concentrations of FOAPs were applied to neuronal cells two hours prior to direct exposure to a β-amyloid protein fragment 25–35 (Aβ25-35). The AD disease model of neuronal cells was developed at cellular level using Aβ25-35 [149]. Polysaccharide fractions significantly inhibited the over accumulation of ROS induced by Aβ25-35 and the release of LDH and MDA, dependent on their intake [149]. FOAPs may also prevent cell apoptosis [149]. Translocation of cytochrome C from mitochondria to the cytosol was decreased, and the Bcl-2/Bax ratio was raised in neuronal cells in response to FOAPs. Moreover, polysaccharide fractions had a neuroprotective effect against Aβ25–35-stimulated cytotoxicity in neuronal cells [149].

#### 2.2.3. Anti-Neuroinflammation Activity of Polysaccharides

Neuroinflammation initiates and enhances neurodegenerative ailments like PD and AD [150]. Microglia and astrocytes protect the brain from infectious agents, while their prolonged activation causes neuroinflammation that can promote neurodegeneration [150]. Currently, there are no treatments to stop the progression of neurodegeneration. Therefore, work is focused on identifying natural compounds that are protective against these diseases [150]. Given that neuroinflammation significantly initiates and enhances neurodegenerative pathology, natural anti-inflammatory compounds may be good candidates for the development of successful therapeutic strategies [150].

Polysaccharides derived from natural sources contain various monosaccharide units joined with each other by several glycoside linkages with a complex molecular arrangement [151]. Polysaccharides have significant pharmaceutical importance due their strong anti-inflammatory and immunomodulatory properties [151]. A raw polysaccharide extracted from *Acorus tatatinowii*, AT50 [14], substantially enhances learning and memory in mice with amnesia caused by scopolamine and inhibits the release of inflammatory mediators, thus could be potentially used in therapy of neurodegenerative disorders. ATP50-3 decreased high levels of inflammatory mediators in lipopolysaccharide (LPS)-induced pro-inflammatory BV2 cells in vitro, as well as inhibiting the stimulation of nuclear factor kappa B (NF-κB) [14]. Furthermore, LPS-induced protein levels of Toll-like receptor 4 (TLR4), protein kinase B (p-Akt), phosphoinositide 3-kinase (p-PI3K) and myeloid differentiation primary response protein (MyD88) were down-regulated by ATP50-3. ATP50-3 protected against neuroinflammation mediated neurological impairment in primary cortical and hippocampal neurons, by alleviating ROS levels and loss of the mitochondrial membrane potential (MMP) [14]. Taken together, these findings indicate that the anti-neuroinflammatory and neuroprotective effects of ATP50-3 are through the TLR4-mediated MyD88/NF-kB and PI3K/Akt signaling pathways [14].

Mediesse et al., investigated polysaccharide *Khaya grandifoliola* fractions (KGF) and *Cymbopogon citratus* fractions (CCF), isolated respectively [152] from stem bark and leaves, for their effect on CNS depression, systemic LPS-induced brain inflammation and hyperalgesia in BALB/c mice. Firstly, the depressant effects of polysaccharide fractions were measured in BALB/c mice weighing around 25–35 g, using the rotarod performance test and an actophotometer [152]. Secondly, one hour after oral administration of polysaccharide fractions (100 mg/kg test dose) or distilled water, LPS or saline solution (5 mg/kg) was intraperitoneally administered. Then, to assess thermal hyperalgesia and brain inflammation, hot plate and tail-flick models were performed one hour post LPS intake and examined by Luminex assay three hours post LPS intake [152]. A complete LPS dose caused a decrease in pain response latency and increased expression of interleukin-1β (IL-1β), IL-6, tumor necrosis factor-α (TNF-α) genes, pro-inflammatory cytokines and NF-κB in the brain after 24 h [152]. Treatment with KGF and CCF (100 mg/kg) decreased LPS-induced hyperalgesia and overexpression of IL-1β, IL-6 and TNF-α genes in the brain dependent on NF-κB signaling [152]. These results suggest that KGF and CCF may have potential as treatments of neuroinflammatory diseases, and that further investigation is required to unravel their exact mechanism of action and dose requirements [152].

Crude polysaccharide AOP70 from *Alpinia oxyphylla* of the ginger family was tested in murine models with induced AD, where it significantly enhanced learning and memory [153], and could be potentially used in therapy of AD. AOP70 decreased the production of NO, prostaglandin E-2 (PGE-2), Interleukin 1 beta (IL-1β), and Tumor necrosis factor (TNF-α) to normal concentrations in the serum of AD affected mice, indicating that polysaccharide considerably enhances memory and learning in diseased mice by anti-neuroinflammation [153]. This crude polysaccharide was purified further to isolate the major constituent, a novel heteropolysaccharide (AOP70-2-1) having a molecular weight of about 76.6 kDa [153]. AOP70-2-1 is a novel acidic polysaccharide with an irregular sheet structure and no triple helical arrangement, with wrinkles on the top surface. After its addition to LPS-modulated BV2 cells, the concentrations of NO, IL-6 and TNF-α decreased significantly [153]. These findings indicate that by preventing the development of pro-inflammatory factors, this polysaccharide could be an active constituent responsible for the anti-neuroinflammatory activities of AOP70. Further clarity is needed to understand the structure-activity relationship, along with mechanistic analyses to understand its anti-neuroinflammatory action [153].

*Schisandra chinensis* whose fruit is called magnolia berry, has been used since ancient times as a medicinal formula for recovery from weak memory or insanity [25]. Xu et al. investigated the action of *Schisandra chinensis* fruit (SCP) polysaccharides on animal models of AD [25]. Immunohistochemistry (IHC) was applied to detect the deposition of Aβ [25]. Certain immune mediators including TNF-α, IL-1β and IL-6 were identified in a specific part of the brain by ELISA [25]. Activation of CNS cells was evaluated via immunofluorescence microscopy. Histopathological modifications were detected by hematoxylin and eosin staining (H&E) [25]. SCP was found to substantially decrease the cognitive and histopathological changes of AD mice, including Aβ accumulation, pro-inflammatory cytokine expression, and activity in brain cells [25]. Furthermore, SCP decreased the phosphorylation of some kinases by displacement to the nucleus [25]. For these reasons SCP could be a potential candidate for the therapy of AD.

Polymannuronate (PM) is an alginate-separated acidic polymer. It is an edible brown algae linear block polysaccharide that is commonly used in the production of food [154]. Seleno-polymannuronate (Se-PM) is a seleno-derivative of PM prepared in the laboratory. The anti-neuroinflammatory role of Se-PM was investigated by Bi et al. [154] in LPS-modulated microglial cells and in an acute inflammatory mouse model. Their findings indicate that this modified polysaccharide could significantly moderate the development of NO and PGE-2 expression and the secretion of interleukins in microglial cells treated with LPS [154]. In addition, Se-PM was attenuated by the LPS-modulated activation of signaling molecules. Furthermore, in vivo, microglial activation induced by LPS was significantly controlled by this PM derivatives [154]. These findings indicate that Se-PM is worthy of further exploration as a functional food to relieve neuroinflammation [154].

Liang et al., investigated the medicinal action of *Dendrobium officinale* polysaccharides (DOPS) on two standard animal models with learning problems and weak memory [155]. Ovariectomy can be triggered by low production of estrogen in mice, and it also causes learning and memory problems [155]. In murine models, D-galactose was subcutaneously provided to induce cognitive impairment [155]. Different techniques such as H&E staining and Nissl staining were applied to investigate the impact of these polysaccharides on hippocampal neurons. Various analytical experiments were performed to explore the impact of polysaccharides on two impaired mice models [155]. In both models, the intake of these polysaccharides substantially ameliorated impaired learning and memory. Additional analyses showed that the polysaccharides control the initiation of Nrf2/HO-1, preventing stimulation of microglia in ovariectomy, D-galactose-stimulated weak cognition, and oxidative damage and neuroinflammation [155]. These results indicate that DOPS has important restorative action on weak learning and memory, and its mode of action may be related to the activation of the Nrf2/HO-1 system, to alleviate oxidative damage and neuroinflammation.

Xu et al. [156] assessed structural features of SCP2-1 polysaccharides obtained from *Schisandra chinensis* (Turcz.) Baill plants and evaluated its anti-neuroinflammatory activity. SCP2-1 had a molar ratio of 8.78:1.23 of glucose to galactose. Evaluation of behavioral pharmacology and biochemical markers indicated that SCP2-1 might ameliorate the cognitive impairment produced by LPS in mice and reduce inflammation [156]. SCP2-1 was shown to decrease the examination period of animals in a novel arm of the Y maze test, reduce the escape latency in the Morris water maze test, and increase the exploration time of the new objects in the NOR test [156]. Treated mice showed improved LPS-induced histopathological changes. They suppressed glial cell over activation, had decreased pro-inflammatory cytokine expression, increased anti-inflammatory cytokine levels, and decreased NLRP3 and M-caspases-1 levels, which can decrease deposition of Aβ [156]. Additionally, the over-activation of NF-κB and hyper-phosphorylation of the P38 MAPK pathway was repressed by SCP2-1. Thus, SCP2-1 should be examined as a potential therapy of AD.

*Polygala tenuifolia* is an industrial-export plant in Southeast Asia, particularly in China [60]. Rhizomes obtained from *P. tenuifolia* are well-known for their cognition enhancing and inotropic properties. They are frequently consumed in traditional Chinese medicine. The functional constituents that account for *P. tenuifolia’s* natural benefits remain elusive. Li et al. [60] used the hot water method to isolate P. tenuifolia rhizomes and purification was performed with Sephacryl S-100 and diethylaminoethyl cellulose (DEAE-C) 52 chromatographic columns. Homogeneous heteropolysaccharide PTP70-2 was obtained with a molecular weight of 65.2 kDa [60]. The pharmacological assessment showed that PTP70-2 repressed nitric oxide production in LPS-induced pro-inflammatory BV2 microglial cells, and the suppressive action of 3.08 μM PTP70-2 was more than that of a positive control (12.5 μM minocycline) [60]. In addition to the inhibition of nitric oxide, the production of pro-inflammatory cytokines such as TNF-α and IL-6 was inhibited by PTP70-2. Based on these observations, the investigators conclude that PTP70-2 is a novel anti-neuroinflammatory agent with the possible capability to ameliorate AD [60].

For hundreds of years, *Ganoderma lucidum* (GL) has been commonly recommended to boost health and longevity in Asian countries [157]. Putative pharmacological functions include the stimulation of innate immunity, cell proliferative control and cancer suppression [157]. Key components of polysaccharides isolated from *Ganoderma lucidum* (GLP) have been suggested previously in the literature to regulate the immune system. A contribution of GLP to neuroinflammation mediated by microglia has not been elucidated [157], and GLP’s effect on microglial behavior is still to be unraveled [157]. Cai et al. [157] thoroughly studied the influence of GLP on BV2 microglia and primary mouse microglia. This quantitative study was performed to find the impact of GLP on microglial pro- and anti-inflammatory cytokine responses, along with behavioral variations such as morphology, movement, and phagocytosis. In the zebrafish brain, study of microglial morphology and modulation of phagocytosis has been verified. GLP downregulated pro-inflammatory cytokines induced by LPS or Aβ and stimulated anti-inflammatory expression of cytokines in BV-2 and primary microglial cells [157]. Furthermore, GLP reduced inflammation-associated microglial movement, variations in morphology, and phagocytosis. The expression of MCP-1 and C1q were also observed in correlation with modulations of microglial behavioral responses [157]. Cai et al. provided insight into GLP-mediated control of neuroinflammation prompted by LPS and Aβ and proposes that the neuroprotective effects of GLP might be provided by modulating inflammatory and behavioral microglial responses.

*Moringa oleifera* is a multi-functional herbal plant used in traditional medicine. Cui et al. [68] obtained a new polysaccharide, known as MRP-1 from Moringa oleifera roots. GC-MS based estimation of monosaccharide configuration found that MRP-1 contained mostly 1.5:2.0:3.1:6.0:5.3:1.1. molar ratios of rhamnose, fructose, arabinose, mannose, xylose, and galactose [68]. Various spectral studies indicated that MRP-1 contained carbohydrate features such as alpha-Araf, β-Galp, α-Gly, β-Gly and α-GalpA [68]. LPS-induced RAW264.7 macrophage cells were selected to determine whether MRP-1 has anti-inflammatory properties. Various doses of MRP-1 stopped LPS-induced TNF-α and NO production [68]. In addition, LPS-induced mRNA expression levels of Inducible nitric oxide synthase (iNOS) were decreased with treatment of multidrug resistance protein 1 (MRP-1), while having no prominent impact on the expression level of COX-2 mRNA [68].

#### 2.2.4. Anticholinesterase Activity of Polysaccharides

Currently, four AChE inhibitors (AChEi) or anti-AChEs, namely donepezil, rivastigmine, galantamine, and memantine, are available for the prevention of dementia and for improving the cognitive deficits of neurodegenerative disorders. AChEi increase the levels of ACh at the synapse and enhance cholinergic activity in the brain [158,159]. The only naturally occurring AChEi with clinical significance is galantamine, an alkaloidal derivative from the *Amaryllidaceae* family of herbal plants [160,161]. Galantamine inhibits ACh reversibly and competitively, and modulates nicotinic ACh receptors allosterically [162,163].

Acetylcholinesterase inhibitors have been used to combat AD [164]. Even so, most of these drugs have undesirable side effects, including dizziness, liver toxicity, bradycardia, and bowel disturbances [164]. As a result, the development of effective anticholinesterase compounds derived from nature is highly anticipated and sought-after.

Natural AChEi have additional pharmacological properties, especially antioxidant properties, making them a multifunctional therapeutic strategy for preventing the occurrence and progression of AD [165,166,167]. Several studies have isolated and identified natural molecules with potential AChEi activity that have shown positive outcomes as novel anti-AD drugs [168]. Natural polysaccharides isolated from natural sources ranging from rice bran to edible mushrooms have shown potential AChEi activity and could be formulated as novel drugs to treat drug-resistant AD.

In 2017, Hafsa and colleagues reported the extraction of some hydrophilic polysaccharides from microalgae such as *Isochrysis galbana* and *Nannochloropsis oculate,* which possessed antimicrobial, anticancer, and anticholinesterase properties [169]. In 2018, Mebrek and colleagues reported that barley-derived beta-glucan, a homopolysaccharide, has moderate antioxidant and enzyme inhibitory activities. In 2017, Pejin and colleagues demonstrated that polysaccharides from two fungal strains, *Coprinus comatus*, and *Coprinellus truncorum*, possessed potential AChEi activity [170]. In 2018, Zhang and colleagues reported that polysaccharides derived from *Flammulina velutipes* had various pharmacological effects, including AChEi activity [171]. In 2019, Deveci and colleagues reported that a polysaccharide obtained from *Pleurotus ostreatus,* a mushroom tree, exhibited significant inhibitory activity against BChE [172]. Badshah and colleagues reported that polysaccharides isolated from a wide range of mushrooms, which contain glucans, krestin, and lentinan, have significant AChE and BChE inhibitory activity and are being regarded as novel drug therapies for the management of AD and PD [173,174]. Also Badshah and colleagues have extracted mushroom polysaccharides from *M. esculenta*. This polysaccharide’s deproteinized form has shown moderate free antioxidant activity but exhibited substantial AChE and BChE inhibitory properties. As a result, these polysaccharides are considered new therapeutic candidates for the treatment of AD and PD [174]. Another class of natural polysaccharides obtained from algae include fucoidans and fucose-containing sulfated polysaccharides such as glucose, mannose, galactose, and uronic acids [175]. These polysaccharides are found to have sulfur complexed with polysaccharides, called sulfated polysaccharides, and they possess high molecular weight and are isolated from *Sargassum horneri* [63]. The most extensively studied sulfated polysaccharides are algae’s fucoidans and sulfated galactans. These sulfated polysaccharides have various physiological and pharmacological actions, including antithrombotic, anticoagulant, antioxidant, anti-inflammatory, antitumor, and immune-modulating properties [176]. The sulfated polysaccharides derived from *Ecklonia maxima, Gelidium pristoides*, and *Ulva rigida* have shown inhibitory action on AChE, BChE, and β-secretase activity. Sulfated polysaccharides induce the elimination of Aβ (1–42) fibrils, which inhibit fibril accumulation, implying that they could have antioxidant and neuroprotective properties in the treatment of AD [15]. In research published in 2019, Rahmani Nezhad and colleagues demonstrated the AChE and BChE inhibitory activities of a broad variety of polysaccharides isolated from two Iranian and French strains of *Agaricus subrufescens*. Both extracts exhibited selective AChE inhibitory action. Furthermore, these extracts had anti-aggregation activity comparable to donepezil [177]. Olasehinde and colleagues recently confirmed that sulfated polysaccharides could prevent apoptosis and necrosis caused by Zn-induced neuronal damage in an AD model. According to them, the neuroprotective effects of sulfated polysaccharides strongly correlate with a reduction in apoptosis, oxidative damage, and AChE activity [18].

Zhang et al. [178] investigated a polysaccharide with antioxidant activity, known as porphyran. This polysaccharide was obtained from the red macroalgae *Pyropia haitanensis*. Findings suggested that it acts as a protective compound against neurotoxicity during AD in mice. Colorimetric methods investigated the action of cholinesterases in hippocampal and cortical tissue [178]. Results showed that porphyran greatly enhanced Aβ1-40 mediated learning and memory impairment [178]. Biochemical research found that porphyran increased choline acetyltransferase activity in hippocampal and cortical tissue and decreased acetylcholinesterase activity. The mechanism may be linked to an increase in the acetylcholine content of the brain. Porphyran has potential as an anti-aging drug [178].

In conclusion, natural polysaccharides appear to have a diversity of functions in neurodegenerative disorders. Hence, they can be developed as a novel class of AChE inhibitory drugs with therapeutic efficacy against a wide range of neurodegenerative disorders, including AD and PD.

#### 2.2.5. Anti-Neurotoxicity Activity of Polysaccharides

Zinc has a prominent role in neuronal signaling and neurotransmission. However, accumulation of zinc in the brain has been linked to PD and AD [179]. Neuroprotective effects have been shown for five different macroalgae to Zn-induced neuronal damage: Ecklonia maxima (KPM), Gracilaria gracilis (GCL), Ulva lactuca (ULT) and Gelidium pristoides (MNP). When provided with aqueous ethanol and zinc sulphate extracts from these seaweeds, cells had reduced apoptosis and acetylcholinesterase activity, as well as altered redox balance involving antioxidant enzymes [149]. Seaweed extracts reduced malondialdehyde and NO levels and prevented zinc-stimulated cell death. In addition, zinc treatment induced a gradual decrease in glutathione levels along with a reduction in the activity of catalase and superoxide dismutase [149]. Macroalgal extract, on the other hand, increased the activity of antioxidant enzymes and glutathione levels. These findings indicate that seaweed extracts improve cholinergic transfer disrupted by zinc-stimulated neurotoxicity [149]. It may be possible to relate the neuroprotective results of macroalgal extracts to their biologically active components. Macroalgae may be significant sources of neuroprotective chemicals for the production of nutraceuticals and other purposeful foods [149].

Olasehinde et al., evaluated the neuroprotective ability against Zinc-stimulated neuronal degeneration of polysaccharide extracted from the five macroalgae Ecklonia maxima (PKPM), Gelidium pristoides (PMNP), Ulva lactuca (PULV), Ulva rigida (PURL) and Gracilaria gracilis (PGCL) [18]. Whereas zinc decreased the viability of cells to around 50%, sulfated polysaccharides, rescued viability to 95% [18]. These sulfated polysaccharides also inhibited zinc-induced necrosis, delayed apoptosis and improved glutathione and antioxidative levels in zinc-treated cells [18].

Li et al. demonstrate the neuroprotective effect of acidic polysaccharide extracted from *Astragalus Membranaceus*, against the neurotoxicity of 6-hydroxydopamine (6-OHDA), which can provoke PD. The isolated membranaceus polysaccharide astragalan exhibited a capability to reduce the degeneration of dopaminergic neurons exposed to roundworm to 6-hydroxydopamine, and increased life span by improving food-sensing behavior. Astragalan also reduced ROS and inhibited lipid peroxidation along with increasing the superoxide dismutase and glutathione peroxidase activity in oxidopamine intoxicated roundworms [180]. Efficiency of membranaceus polysaccharides in reducing the transcript level of proapoptotic gene egl-1 in oxidopamine exposed roundworms was also reported. Based on these findings the membranaceus polysaccharide astragalan may suppress oxidopamine neurotoxicity by eliminating oxidative stress, and rehabilitate cholinergic system function and regulate apoptotic proteolytic enzymes.

AD gradually and progressively destroys nerve tissue, and has a negative impact on memory and cognitive function, leading to confusion and disorientation in space and time. AD affects mostly individuals over 60, even if AD early-onsets can be diagnosed in younger individuals [181]. A series of epidemiological and clinical investigations have demonstrated that high plasma homocysteine levels increase the likelihood of AD. Although the biological mechanisms of its toxicity remain unclear, it has been shown that homocysteine induces DNA fragmentation, neuronal apoptosis, and tau hyperphosphorylation in neurons. Earlier investigations showed that polysaccharides derived from Lycium barbarum (LBA) have the capacity to protect nerve cells from Aβ peptide neurotoxicity. Neuroprotective effects of polysaccharides isolated from *Lycium barbarum* may not be limited to Aβ peptide and may also further protect against other AD risk factors [182]. Polysaccharides isolated from *Lycium barbarum* significantly reduced homocysteine induced nerve cell death and apoptosis in primary cortical neurons as measured by caspase-3 like activity and the lactate dehydrogenase (LDH) test. Also, polysaccharides isolated from *Lycium barbarum* drastically reduced the effect of homocysteine on tau phosphorylation at tau-1 (Ser198/199/202), pS214 (Ser214) and pS396 (Ser396), as well as deprotection of tau. The overall data collected from this work demonstrates the ability of the polysaccharides to reduce the risk of AD [51].

#### 2.2.6. Polysaccharides as Ferroptosis Switchers

Ferroptosis induced cell death is recognized as one of the leading processes in neurodegeneration [183]. Consequently, naturally occurring inhibitors of ferroptosis rise growing attention of scientists as therapeutic candidates in the treatment of neurodegenerative diseases [184]. Surprisingly, among different class of molecules examined as ferroptosis inhibitors for neurodegenerative disorders, polysaccharides seem to be omitted and has not been examined.

Different dose-dependent natural products can increase intracellular reactive oxygen species (ROS) and unbalance redox homeostasis, for instance flavonoids, glycoside, polysaccharides, which are promising molecules for ferroptosis induction. For example, solanine, a glycoside present in potatoes, tomatoes, and eggplant, has been shown to decrease glutathione (GSH) levels by enhancing the ROS production in HepG2 cells [185]. Reduced GSH synthesis can indirectly affect the activity of GPX4, and it is reasonable to hypothesize that solanine-induced cell death at least partially involves ferroptosis [186].

Gallic acid (GA) is a natural anti-cancer compound that can be found in many food sources, e.g., edible mushrooms, fruits, and vegetables. Gallic acid and its derivatives, namely theaflavin-3-gallate and theaflavin-3′-gallate, have been proven to initiates ferroptosis in HeLa cell lines, by reducing intracellular GSH, increasing the production of ROS, and promoting lipid peroxidation [187]. Pyrogallol [188] and propyl gallate [189] can also reduce the content of GSH, promote the ROS production, and most probably become new inducers of ferroptosis. Tang and Cheung [190] showed that GA can activate iron-dependent cell death with apoptotic, ferroptotic, and necroptotic features. Their time-lapse live-cell microscopy experiments demonstrated that GA could induce contemporarily different RCD types, including apoptosis with mitochondrial cytochrome c release and caspase-3 activation, ferroptosis with lipid peroxidation, and necroptosis with the loss of plasma membrane integrity. Of note, GA-induced cell death could be totally suppressed by the administration of deferoxamine-known iron chelator.

Astragalus polysaccharide (APS) is an active ingredient of *Astragalus membranaceus* and has been shown to ameliorate experimental colitis—an inflammatory bowel disease (IBD). Chen et al. [191] observed that APS administration in experimental colitis mice APS attenuated total weight loss, colon length shortening, histological damage, and the expression of inflammatory cytokines in the colon. Such ameliorations were correlated with ferroptosis inhibition, by the decrease in the expression of ferroptosis-associated genes (PTGS2, FTH, and FTL) and the levels of surrogate ferroptosis markers (Malondialdehyde (MDA), GSH, and iron load). Mechanistically, the inhibitory effects of APS on ferroptosis were associated with the NRF2/HO-1 pathway.

## 3. Conclusions

Polysaccharides is a heterogeneous class of macromolecules with distinct properties depending on their monosaccharide building blocks and multiple functional groups. Polysaccharides are an important class of biological polymers, which function in living organisms has been associated strictly to structure- or storage-related functions. Surprisingly, growing number of recent studies showed that polysaccharides participate number of biological intra- and extracellular processes with antioxidant, anticoagulant, antithrombotic, anticancer and many other effects. Inhibitory potential of polysaccharides in the inflammation, oxidation, regulated cell death and Acetylcholinesterase enzymatic activity can be translated into therapeutic use of polysaccharides in neurodegenerative disorders. In this review, we presented different molecular mechanism involved in neurodegeneration and how numerous polysaccharides of natural origin can inhibit such processes.

## Figures and Tables

**Table 1:** Summary of neuroprotective and anti-neurodegenerative properties of natural polysaccharides described in scientific literature.

Polysaccharide	Class (Origin)	Effect (Activity)	Model or Technique	Reference
Sulfated polysaccharides (Cr-SPs)	Algae (*Chlamydomonas reinhardtii*)	Preventive (Anti-PD activity; α-Synuclein fibrillation inhibition)	Fluorescence kinetics and 90° light scattering studies	[20]
Sulphated polysaccharides (Cr-SPs)	Algae (*Chlamydomonas reinhardtii*)	Preventive (Anti-PD activity)	Fibrillation/aggregation of α-Syn mutants by spectroscopic and microscopic techniques	[21]
Sulphated agaran	Algae (*Gracilaria cornea*)	Preventive (Anti-PD activity)	Rat model of PD induced by 6-hydroxydopamine	[19]
Polysaccharide (APS)	Plant (*Astragalus*)	Preventive (Anti-PD activity; activates autophagy)	PC12 cells induced by 6-HODA	[22]
Polysaccharide (IOPS)	Fungi (*Inonotus obliquus*)	Preventive (Anti-AD activity)	HT22 mouse hippocampal neuronal cells	[23]
Purified polysaccharide (CYP)	Plant (*Corydalis yanhusuo*)	Preventive (Neuroprotective effects on Aβ (25–35)-induced neurotoxicity)	PC12 induced by 6-OHDA	[28]
Sulfated fucans	Algae (Five brown algae)	Preventive (Anti-AD activity)	Vero cells infected with HSV1	[29]
Polysaccharides (POP)	Fungi (*Pleurotus oystreatus*)	Preventive (Anti-AD activity)	Male Wistar rats	[30]
Polysaccharides (PTM)	Plant (*Taxus chinensis var. mairei Cheng et L.K.Fu (Taxaceae)*)	Preventive (Anti-AD activity and anti-neurotoxicity)	C57BL/6 mice	[31]
Polysaccharides (CPPs)	Plant (*Codonopsis pilosula*)	Preventive (Anti-AD activity)	APP/PS1 mice	[32]
Polysaccharides (LBP1)	Plant (*Lycium barbarum*)	Preventive/Therapeutic (Anti-AD activity)	APPswe/PS1ΔE9 (APP/PS1) transgenic mice	[33]
Polysaccharide-enriched aqueous extract (HE)	Fungal (*Hericium erinaceus*)	Preventive (Anti-AD activity)	PC12 cells	[34]
Dermatan sulfate	Animal (*Ascidian Phallusia nigra*)	Preventive (Antioxidant activity and neuroprotection)	Neuro-2A cell lineage	[35]
Polysaccharide (CPA-1 and CPB-2)	Fungal (*Cordyceps cicadae*)	Preventive (Antioxidant activity)	PC12 cells	[36]
Sulfated polysaccharides (CFCE-PS)	Algae (*Seaweed Codium fragile*)	Preventive (Antioxidant activity)	Zebrafish embryos	[37]
Fucoidan	Algae (*Dictyota ciliolate*)	Preventive (Antioxidant activity)	HepG2 cells	[38]
Fucoidan	Algae (*Ecklonia cava*)	Preventive (Antioxidant activity)	AAPH-induced oxidative stress in zebrafish	[39]
Fucoidan	Algae (*Marine brown algae*)	Preventive (Antioxidant activity)	human keratinocyte cell line (HaCaT)	[40]
Fucoidan	Algae (*Undaria pinnatifida*)	Preventive/Therapautic (Neuroprotective activity)	D-Gal-induced neurotoxicity in PC12 cells and cognitive dysfunction in Mice	[41]
Fucoidan	Algae (*Turbinaria decurren*)	Preventive/Therapautic (Neuroprotective activity)	MPTP-treated C57BL/6 mice	[42]
Fucoidan	Algae (*Marine brown algae*)	Preventive/Therapautic (Neuroprotective activity)	Cerebral ischemia reperfusion injury Sprague-Dawley rats	[43]
Fucoidan	Algae (*Sargassum crassifolium*)	Preventive/Therapautic (Neuroprotective activity)	H2O2-treated PC-12 cells	[44]
Fructan	Plant (*Anemarrhena asphodeloides*)	Preventive/Therapautic (Neuroprotective and immunoregulatory effects)	SH-SY5Y cell injury model	[45]
Polysaccharide (APS)	Plant (*Astragalus*)	Preventive/Therapautic (Neuroprotective activity)	Mouse PD model	[46]
Polysaccharide (ALP)	Plant (*Annona muricata*)	Preventive/Therapautic (Neuroprotective activity)	H_2_O_2_-treated mouse hippocampal neuronal cells (HT22)	[47]
Heteropolysaccharide SV2-1	Animal (Squid)	Preventive/Therapautic (Neuroprotective activity)	PC12 induced by 6-OHDA	[48]
Ulvan	Algae (*Chlorella pyrenoidosa*)	Preventive/Therapautic (Neuroprotective activity)	MPTP-treated C57BL/6J mice	[49]
Polysaccharides (PSP)	Algae (*Spirulina platensis*)	Preventive/Therapautic (Neuroprotective activity)	MPTP-treated C57BL/6J mice	[50]
Polysaccharides (LBA)	Plant (*Lycium barbarum*)	Preventive/Therapautic (Neuroprotective activity)	Rat cortical neurons	[51]
Heteropolysaccharide (LFP-1)	Plant (*Lycii fructus*)	Preventive/Therapautic (Neuroprotective activity)	Highly differentiated PC12 cells	[52]
Polysaccharides (SCP)	Fungal (*Sparassis crispa*)	Preventive/Therapautic (Neuroprotective activity)	Immortalized mouse hippocampal cell line HT22	[53]
Polysaccharides (GLPS)	Fungal (*Ganoderma lucidum*)	Preventive/Therapautic (Neuroprotective activity)	Adult male Wistar Albino rats with a mean age of 8 months	[54]
Polysaccharides (GPP1)	Plant (*Gynostemma pentaphyllum*)	Preventive/Therapautic (Neuroprotective activity)	PC12 cells	[55]
Polysaccharides (MAPs)	Plant (*Opuntia Milpa Alta*)	Preventive/Therapautic (Neuroprotective activity)	Cultured cortical neurons	[56]
Crude polysaccharide fraction (DEVP)	Fungal (*Dictyophora echinovolvata*)	Preventive/Therapautic (Neuroprotective activity againts hydrogen peroxide-induced toxicity	PC12 cells	[57]
Polysaccharides (PEPF)	Plant (*Perilla frutescens*)	Preventive/Therapautic (Neuroprotective activity)	HT22 cells, mouse hippocampal neuronal cell lines	[58]
Selenium polysaccharides	Plant (*Radix hedysari*)	Preventive/Therapautic (Neuroprotective activity)	SH-SY5Y cells	[59]
Heteropolysaccharide (PTP70-2)	Plant (*Polygala tenuifolia*)	Preventive/Therapautic (Anti-neuroinflammatory activity)	BV2 microglial cells	[60]
Sulfated polysaccharide (GLPss58)	Fungal (*Ganoderma lucidum*)	Preventive/Therapautic (Anti-neuroinflammatory activity)	C57BL/6 mice, BMMs, MSLs, HPBLs	[61]
Polysaccharides (ALP-1)	Plant (*Arctium lappa*)	Preventive/Therapautic (Anti-neuroinflammatory activity)	DSS-induced colitis in ICR mice	[62]
Fucose-rich sulfated Polysaccharides (F4 fraction)	Algae (*Sargassum horneri*)	Preventive/Therapautic (Anti-neuroinflammatory activity)	LPS-stimulated RAW 264.7 cells and LPS-treated zebrafish embryos	[63]
Polysaccharides (PSC)	Plant (*Sargentodoxa cuneata*)	Preventive/Therapautic (Anti-neuroinflammatory activity)	LPS-stimulated RAW264.7 cells and Carrageenan-induced edema in male SD rat paws	[64]
Ulvan	Algae (*Ulva intestinalis*)	Immunostimulation	J774A.1 cell	[65]
Alkali-soluble polysaccharides (ASPP)	Plant (Purple sweet potato)	Preventive/Therapautic (Anti-neuroinflammatory activity)	LPS-treated RAW 264.7 macrophage cells and mice	[66]
Polysaccharide (PLS)	Plant (*Thuja occidentalis Linn*)	Preventive/Therapautic (Anti-neuroinflammatory activity)	Carrageenan (or other agents)-induced paw edema model in male Swiss mice	[67]
Polysaccharide (MRP-1)	Plant (*Moringa oleifera roots*)	Preventive/Therapautic (Anti-neuroinflammatory activity)	LPS-induced RAW264.7 macrophages	[68]
Polysaccharides (WSRP-1b)	Plant (*Kushui rose*)	Preventive/Therapautic (Immunomodulatory activity)	RAW264.7 cell line	[69]
Polysaccharide (AFP-2)	Plant (Apios americana)	Preventive/Therapautic (Anti-neurotoxicity)	PC12 cells	[70]
Polysaccharide (EbPS-A1)	Plant (*Epimedium brevicornum*)	Preventive/Therapautic (Anti-neurotoxicity)	Caenorhabditis elegans and Escherichia coli strains	[71]
Polysaccharides (LBP)	Plant (*Lycium barbarum*)	Preventive/Therapautic (Anti-neurotoxicity)	PC12 cells	[72]
Laminarin	Algae (Brown seaweed)	Preventive/Therapautic (Neuronal activation)	Gerbils	[73]
Fucoidan	Algae (Laminaria Japonica)	Preventive/Therapautic (Counteracts memory deficits)	Sprague–Dawley rats	[74]
Polysaccharide (LRP3)	Plant (*Lycium ruthenicum*)	Preventive/Therapautic (Neuronal activation)	Sprague-Dawley (SD) rats	[75]
Polysaccharides (MCPs)	Plant (*Momordica charantia*)	Preventive/Therapautic (Promotes neuronal activation)	C17.2 cells, an immortalized NSC line	[76]
Polysaccharide (DPRG)	Fungal (*Phellinus ribis*)	Preventive/Therapautic (Neurotrophic activity)	PC12 cells	[77]

## Abbreviations

Acetylcholinesterases (AChE); AChE inhibitors (AChEi); Alpinia oxyphylla polysaccharide (AOP); Alzheimer’s disease (AD); Amyloid beta (Aβ); amyotrophic lateral sclerosis (ALS); Astragalus polysaccharide (APS); butyrylcholinesterase (BChE); central nervous system (CNS); chitosan (CHT); cholinesterases (ChEs); crude extract (CE); Cymbopogon citratus fractions (CCF); Dendrobium officinale polysaccharides (DOPS); diethylaminoethyl cellulose (DEAE-C); 3-(4,5-dimethylthiazol-2-yl)-2,5-diphenyltetrazolium bromide (MTT); dopamine transporters (DAT); Ecklonia maxima (KPM); exopolysaccharides (EPSs); Fomes officinalis Ames polysaccharides (FOAPs); free sugar (FS); Gallic acid (GA); Ganoderma lucidum (GL); Ganoderma lucidum polysaccharide (GLP); Gelidium pristoides (MNP); glutathione (GSH); Glutathione Peroxidase 4 (GPX4); Gracilaria gracilis (GCL); high molecular weight (HM); Huntington’s disease (HD); Immunohistochemistry (IHC); Inducible nitric oxide synthase (iNOS); inflammatory bowel disease (IBD); Interleukin 1 beta (IL-1β); Khaya grandifoliola fractions (KGF); lactate dehydrogenase (LDH); lipopolysaccharide (LPS); low molecular weight (LM); Lycium barbarum polysaccharides (LBP1); mitochondrial membrane potential (MMP); multidrug resistance protein 1 (MRP-1); myeloid differentiation primary response protein (MyD88); nitric oxide (NO); noradrenaline transporters (NAT); N-trimethyl chitosan chloride (TMC); nuclear factor kappa B (NF—κB); Parkinson’s disease (PD); phlorotannin (PT); phosphoinositide 3-kinase (p-PI3K); Polygala tenuifolia polysaccharide (PTP); Polymannuronate (PM); polysaccharide (PS); polysaccharides (PS); prostaglandin E-2 (PGE-2); protein kinase B (p-Akt); proteo-β-glucan (PGM); reactive oxygen species (ROS); regulated cell death (RCD); Schisandra chinensis polysaccharides (SCP); Seleno-polymannuronate (Se-PM); sulfated polysaccharides (SPS); Toll-like receptor 4 (TLR4); transmissible spongiform encephalopathies (TSEs); tris(2-carboxyethyl) phosphine (TCEP); Tumor necrosis factor (TNF-α); Ulva lactuca (ULT).
